# Preparation and Performance Investigation of Epoxy Resin-Based Permeable Concrete Containing Ceramsite

**DOI:** 10.3390/polym15244704

**Published:** 2023-12-14

**Authors:** Shengjia Li, Honghai Cui, Haihua Wang, Wensheng Wang, Yi Sui, Liangyu Dong, Jiaxing Wang

**Affiliations:** 1 Guangxi Xinfazhan Communication Group Co., Ltd., Nanning 530029, China; lisj72@outlook.com; 2 College of Transportation, Jilin University, Changchun 130025, China; suiyi1721@mails.jlu.edu.cn (Y.S.); dongly1721@mails.jlu.edu.cn (L.D.); wangjx1721@mails.jlu.edu.cn (J.W.); 3 Jilin Traffic Planning and Design Institute, Changchun 130021, China; cuihh23@outlook.com; 4 Guangxi Transportation Science and Technology Group Co., Ltd., Nanning 530007, China

**Keywords:** permeable concrete, epoxy resin, ceramsite, performance analysis, simulation exploration

## Abstract

Permeable concrete is an innovative type of concrete that provides a sustainable solution for stormwater management by allowing water to seep through and be filtered naturally. This study focuses on the preparation and performance investigation of an epoxy resin-based permeable concrete containing ceramsite. In this study, ceramsite, a lightweight aggregate, is used as a substitute for conventional aggregates in the concrete mixture. The epoxy resin is then added to improve the strength and durability of the concrete. A series of tests, including compressive strength, water permeability, and freeze-thaw resistance tests, are conducted to evaluate the performance of the epoxy resin-based permeable concrete. The results show that with an increasing epoxy resin binder–aggregate ratio, the compressive strength of the epoxy resin-based permeable concrete significantly increases while the permeability coefficient decreases. Different types of aggregates have varying effects on the compressive strength and permeability coefficient of epoxy resin-based permeable concrete, with high-strength clay ceramsite providing the highest compressive strength and lightweight ceramsite having the highest permeability coefficient. In addition, the discrete element simulation method effectively and feasibly determines the ultimate load and accurately simulates the compressive strength of the permeable cement-based mixture, consistent with the measured compressive strength. A quadratic polynomial regression analysis yielded an R^2^ value of around 0.93, showing a strong relationship between durability and freeze-thaw cycles. The findings contribute to the development of sustainable construction materials for stormwater management and offer potential applications in various infrastructure projects.

## 1. Introduction

Permeable concrete, referred to as permeable pavement, is a type of concrete with a high porosity that allows water to pass through it [1,2,3,4]. This innovative construction material has gained significant attention in recent years due to its ability to minimize rainwater runoff as well as enhance its quality. It also helps to replenish groundwater supplies and ameliorate the consequences of urban heat islands [5,6,7,8].

One promising approach to creating permeable concrete is through the incorporation of ceramsite, a lightweight aggregate material derived from clay minerals [9,10,11]. Ceramsite offers several advantages such as a high porosity, low density, and good thermal insulation properties, making it an ideal candidate for use in permeable concrete applications [12,13]. There are many studies on the use of ceramics as substitute aggregates for concrete materials. Researchers test and analyze the physical properties of ceramics such as the granule outline, granule dimension, SSA, etc., to understand their performance characteristics in concrete [14]. At the same time, the chemical composition, compressive strength, water absorption, etc., of ceramics are also studied to determine their applicability and influencing factors in concrete [15]. In order to achieve optimal concrete performance, researchers need to determine the blending ratio of ceramics in concrete. By conducting blending tests with different ratios of ceramics, researchers evaluate the mechanical properties, compactness, durability, and other indicators of concrete to determine the optimal blending ratio [16,17]. Additionally, they study the resistance of ceramics in concrete to sulfate erosion, chloridion erosion, freeze-thaw cycling, etc., to evaluate the durability of ceramics in concrete [18,19]. They also explore the factors and measures that may lead to diminution in a ceramic mixture’s endurance. The current research has achieved some results, indicating that ceramics as substitute aggregates for concrete materials have certain potential. Ceramics can reduce the compactness of concrete, improve the elasticity modulus, and enhance its crack resistance. In addition, the inclusion of ceramics can reduce the thermal shrinkage and drying shrinkage of concrete and improve its durability [20,21]. However, there are still some challenges in the current research, such as the weak interface bonding between ceramics and the cementitious matrix, and the impression of ceramics on the mechanics as well as the enduringness of concrete [22]. Therefore, further in-depth research and experiments are needed to better apply ceramics in concrete materials.

Epoxy resin, as an important thermosetting resin, is popularly applied in various areas; for instance, in adhesives, civil engineering materials, aerospace materials, electronic machinery, electrical insulation materials, and various composite materials [23,24,25]. It has the characteristics of a strong bonding force, high physical and mechanical strength, chemical resistance, durability, excellent process performance, good high-temperature stability, low curing shrinkage rate, and excellent electrical insulation performance [26,27,28]. Epoxy resin is a commonly used binder in the preparation of permeable concrete. It acts as the adhesive, binding the aggregate materials together and providing structural stability. An epoxide resin-based mixture material is a fresh mixture type that uses epoxy resin as a binding material, and it is widely used in important engineering projects such as bridges, tunnels, and ports [29,30]. Researchers modify concrete by adding different types of epoxy resins to improve its mechanical properties, durability, and crack resistance. Currently, common modification methods include adding epoxy resin coatings, preimpregnation, and encapsulation, which can improve the mechanics as well as endurance for concrete [31]. The interface bonding mechanism is one of the key issues in epoxy resin-based concrete materials [32]. Therefore, researchers study the physical and chemical interaction mechanisms between epoxy resins and concrete through experiments and numerical simulations, exploring the influencing factors and improvement methods of interface bonding [33]. Epoxy resin-based concrete materials have been widely used in the engineering of bridges, tunnels, and underground structures. Through practical engineering applications, researchers evaluate and verify the performance of epoxy resin-based concrete materials under different engineering conditions and propose corresponding engineering design and construction requirements.

The objective of this investigation is to investigate the preparation and performance of epoxy resin-based permeable concrete containing ceramsite. The specific objectives include evaluating the mechanics, permeability, as well as enduringness for the resultant concrete. The research will contribute to the development of sustainable infrastructure solutions by providing a better understanding of the potential of epoxy resin-based permeable concrete containing ceramsite. The findings will contribute to the enhancement of sustainable construction practices, particularly in the field of stormwater management and urban water infrastructure. By reducing stormwater runoff and improving water quality, this research can contribute to the overall sustainability of urban environments and help mitigate the adverse impacts of climate change.

## 2. Materials and Methods

### 2.1. Raw Materials

As an essential constituent of permeable concrete, the quality of the cementitious materials has a direct effect on the compression stress and frost resistance for said concrete. In this particular investigation, a modified epoxy resin binder is employed as the cementitious material. This binder is characterized as a solvent-free, two-component product with a nonirritating odor, as well as being environmentally friendly. Epoxy resin is classified as a thermosetting resin that exhibits several advantageous traits post-curing and polymerization including high strength, excellent adhesion, corrosion resistance, and favorable mechanical properties. Table 1 presents the physical properties for the modified epoxide resin binder, which are similar to the technical properties in the previous study [34,35]. The viscosity of the epoxide resin utilized falls within a range of 11,000–14,000 mPa·s, whereas the curing agent possesses a viscosity of 1000–1300 mPa·s.

Aggregates are essential constituents of permeable concrete and serve as load-bearing materials. The compression stress of the epoxy resin-based permeable mixture is significantly influenced through aggregate intensity. Therefore, the selection of aggregates must carefully consider both their permeability and strength. In this study, various types of aggregates were chosen for permeable concretes. One type was an ordinary broken rock aggregate with a particle scale 9.5 mm, while the remaining type was ceramsite. The ceramsite comprises low-weight ceramsite with a particle scale 13.2~16 mm and high-strength clay ceramsite with a particle scale 8 mm. The relevant physical characteristics for these categories of aggregates are presented in Table 2.

### 2.2. Mix Design and Specimen Preparation of Epoxy Resin-Based Permeable Concrete

**Figure 1:**
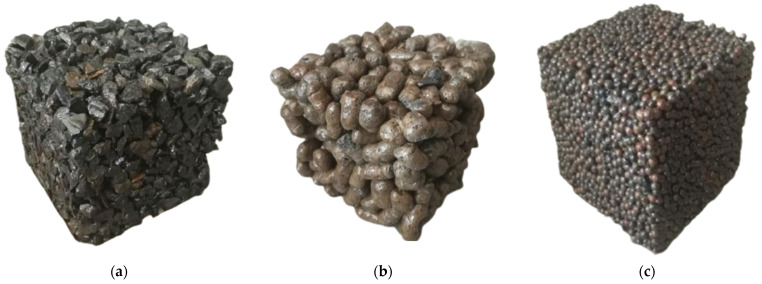
The epoxy resin-based permeable mixtures: (**a**) normal broken rock; (**b**) low-weight ceramsite; and (**c**) high-strength clay ceramsite.

In this study, the epoxy resin binder was utilized as a substitute for conventional cement binder as a cementitious material. However, there were no specific experimental procedures or design standards followed during the mix design process. With reference to cement-permeable concrete, the amount of epoxy resin binder for epoxy resin-based permeable concrete could be determined through trial mixing while keeping the aggregate amounts constant. The epoxy resin binder, being a viscous liquid, uniformly adhered to the aggregate surfaces without any accumulation or dryness. The final mixtures for epoxy resin-based permeable concrete referred to the previous study [18], in which three binder–aggregate ratios were set for each permeable concrete with different aggregate types. The pervious concrete mix proportioning data is shown in Table 3, where the epoxy resin binder–aggregate ratio is the ratio of epoxy resin-based cementitious material to the aggregate by weight. Figure 1 illustrates the specimens of epoxy resin-based permeable concrete with 3 categories for aggregates: normal broken rock, low-weight ceramsite, and high-strength clay ceramsite.

### 2.3. Experimental Tests

In this investigation, the compressive strength examination of porous epoxy resin-based concrete specimens was carried out using an electro-hydraulic universal testing machine, in accordance with Chinese specifications (GB/T 50081-2002) [36]. In the preceding compression experiment, both the porous epoxy resin-based mixture and the top as well the bottom stress flat surfaces for examining equipment were thoroughly erased as well as decontaminated. The core of the porous epoxy resin-based mixture was aligned with the core of the stress flat surface, ensuring that the top surface of the porous epoxy resin-based mixture remained parallel with the stress flat surface throughout the testing procedure. Compression stress examining procedures necessitated a continuous and stable application of load on the examining equipment at a loading velocity of 2 mm/min. Any sudden pressure drops experienced by the testing machine necessitated the immediate unloading of the specimen, with the maximum pressure recorded during this event representing the failure load of the porous epoxy resin-based concrete. Furthermore, for the same concrete mix, the average of 3 samples conducted on the porous epoxy resin-based mixture was calculated.

To assess the permeability coefficient of porous concrete, two methods are employed: a constant-head test technique and variable-head test technique. During the investigation, adhering to Chinese specifications (CJJ/T 135-2009) [37], a variable-head test technique was adopted to fabricate a penetrability meter towards the porous epoxy resin-based mixture employing organic glass. The penetrability coefficient is quantified by a liquid flux density for porous concrete samples within a given time period. Prior to determining the permeability coefficient, the permeability meter underwent calibration using various scales. The permeability meter was then placed onto the porous concrete samples and the connection was sealed. Subsequently, approximately 600 milliliters of liquid were introduced into a penetrability testing instrument, and a liquid shutdown result was swiftly removed. Commencing timing when the liquid exterior lowered nearly 100 milliliters, the timing ceased when the liquid exterior decreased nearly 500 milliliters, acquiring its liquid penetrability characteristics.

**Figure 2:**
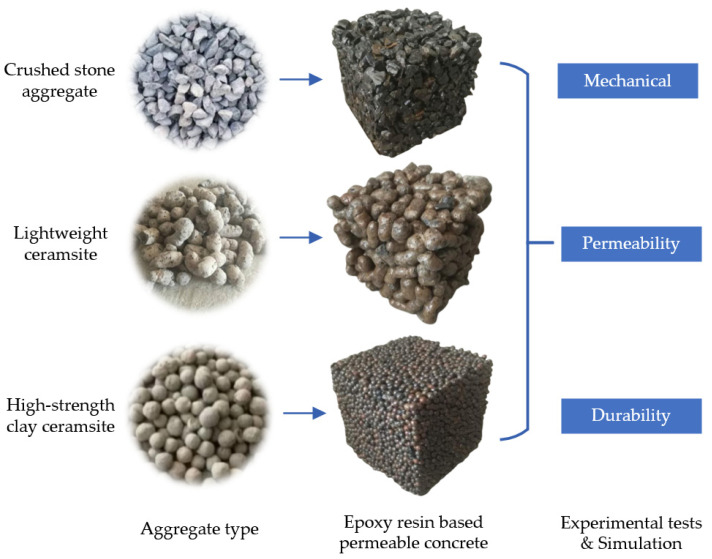
Research graph of the investigation.

Porous epoxy resin-based mixtures were extracted from a curing chamber and immersed into water of 20 °C. A water level must be maintained at a height of 2 cm to 3 cm above the specimens to ensure complete saturation of the porous concrete. After a period of 3 days, these mixture samples were removed from liquid and exposed to freeze-thaw loop experiments specifically designed for porous concrete, following the guidelines outlined in the Chinese specification (GB/T 50082-2009) [38]. The freeze-thaw loop experiment involved measuring the compression stress as well as weight for porous epoxy resin-based mixtures at various stages, including prior to the test and after five, fifteen, and twenty-five freeze-thaw loops. The evaluation of freeze-thaw loop endurance for specimens relied on an assessment of mass-loss rates as well as compression stress-loss rates. The freeze-thaw loop experiment, as per Chinese specifications (GB/T 50082-2009), would continue until one of the following conditions is met: (1) the completion of a predetermined amount of freeze-thaw loops, (2) a compression stress attrition rate of twenty-five percent is reached, or (3) a weight attrition rate of five percent is reached. Figure 2 plots an exploration graph of the investigation.

## 3. Results and Discussion

### 3.1. Compression Stress Analysis of Epoxy Resin-Based Permeable Concrete Considering Aggregate Type

**Figure 3:**
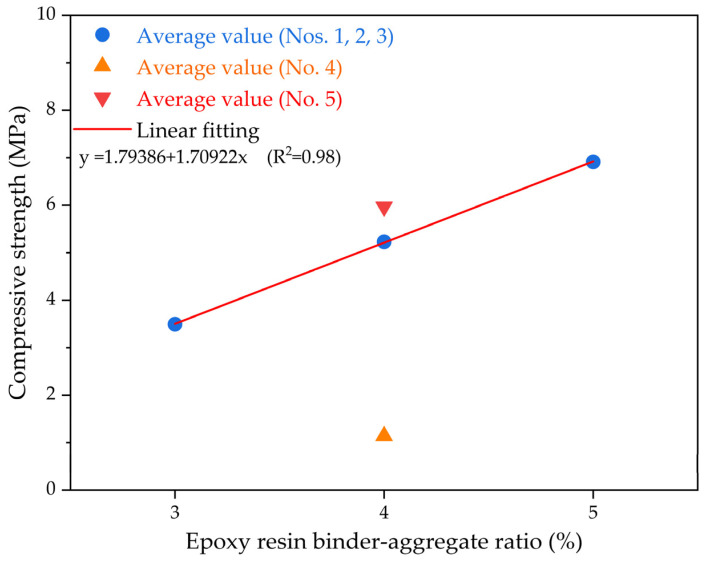
The compression stress results of epoxy resin-based permeable concrete under different epoxy resin binder–aggregate ratios.

Figure 3 depicts the impact of the epoxy resin–aggregate proportion on seven-day compressive stress for the epoxide resin-based permeable concrete using a crushed stone aggregate. As shown in Figure 3, as the epoxy resin–aggregate ratio increases, there is generally a noticeable increase in the compression stress for epoxide resin-based permeable concrete with a crushed stone aggregate. Since epoxy resin-based permeable concrete specimens primarily consist of two components, namely aggregates and an epoxy resin binder, while keeping the amount of the epoxy resin binder, aggregate, and aggregate particle size constant, an amplification in the epoxy resin binder–aggregate ratio brings about a gradual growth for the cementitious binder amount in epoxide resin-based permeable concrete specimens. The strength of permeable epoxy resin-based mixtures incorporating broken rock aggregates primarily relies on the properties of the aggregates as well as the adhesion quality between the epoxide resin binder and aggregates. The epoxy resin binder–aggregate proportion straightly influences the thickness as well as the adhesion region for the cementitious binder on the aggregate exterior. Figure 3 demonstrates that for various groups of epoxy resin-based permeable concrete specimens with different epoxy resin binder–aggregate proportions, the aggregate amount as well as the granule scale of the permeable epoxy resin-based mixture incorporating broken rock aggregates remain constant. The intensity primarily relies on the epoxy resin binder, and as the epoxy resin binder–aggregate ratio increases, the epoxy resin binder layer between the aggregates gradually becomes thicker. Additionally, because of a comparatively increased percentage of cementitious binder composites, the adhesion quality of the epoxy resin binder sheet as well as the aggregate is also enhanced. Consequently, an increasing epoxy resin binder–aggregate ratio corresponds to an overall expansion in compression stress for the epoxy resin-based permeable concrete with crushed stone aggregates. A linear regression analysis was employed to quantitatively appraise the association between the epoxy resin binder–aggregate proportion and the compression stress of epoxy resin-based permeable concrete. It is evident that there is a strong correlation between the epoxy resin binder–aggregate proportion and compression stress, with an R^2^ value of approximately 0.98.

**Figure 4:**
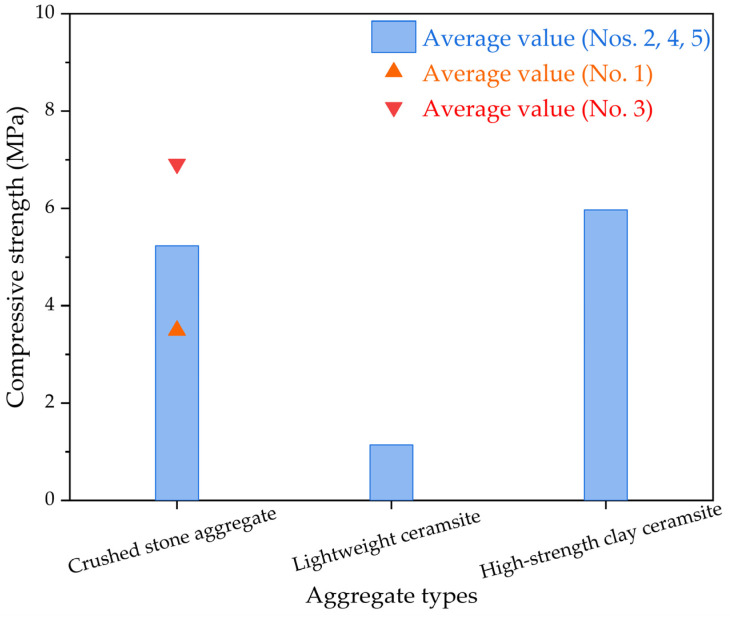
The compression stress results of permeable epoxy resin-based mixture incorporating various categories of aggregates.

The seven-day compression stress results for the permeable epoxy resin-based mixture with various types of aggregates are depicted in Figure 4 via compression stress tests. The compression stress for epoxy resin-based permeable concrete primarily relies on the aggregate strength, with the disparity in compression stress for samples reflecting the varying strength of the selected aggregates. Similarly, some researchers found that the type and particle size of aggregates have an impact on the mechanical, physical, and permeability properties of permeable concrete [39,40]. In epoxy resin-based permeable concrete specimens, a portion of the cementitious binder contributes to binding the aggregates during the formation process, while another portion is found between aggregates, delivering a specific intensity upon curing. As for the permeable epoxy resin-based mixture incorporating various categories of aggregates, the seven-day compression stress results follow the following sequence: high-strength clay ceramsite > normal broken rock aggregate > low-weight ceramsite.

### 3.2. Permeability Coefficient Analysis of Epoxy Resin-Based Permeable Concrete Considering Aggregate Type

**Figure 5:**
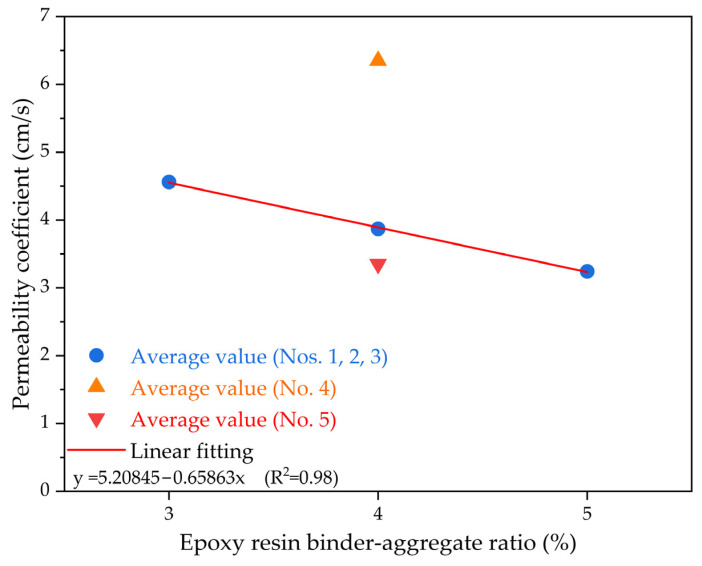
The permeability coefficient results of permeable epoxy resin-based mixtures under various epoxy resin binder–aggregate proportions.

Figure 5 demonstrates the impact of the epoxy resin binder–aggregate proportion on the permeability coefficient for epoxy resin-based permeable concrete. The permeability coefficient gradually decreases as the epoxy resin binder–aggregate ratio increases. Conforming to the composite design presented in Table 3, the aggregate quantity as well as the granule scale for epoxy resin-based permeable concrete with crushed stone aggregates remain constant for various groups of specimens with various epoxy resin binder–aggregate proportions. With a growth in the epoxy resin binder–aggregate proportion, the amount of cementitious binder utilized in the epoxy resin-based permeable concrete specimens gradually rises, leading to an increased volume of cementitious binder materials. This, in turn, results in a reduction in the porosity of the aggregates within the epoxy resin-based permeable concrete specimens, and subsequently decreases the penetrability coefficient. Furthermore, a linear regression analysis was employed to quantitatively evaluate the association of the epoxy resin binder–aggregate proportion and the permeability coefficient for epoxy resin-based permeable concrete. It can be observed that there is a strong correlation (R^2^ ≈ 0.98) between the parameters of epoxy resin binder–aggregate ratio and penetrability characteristics.

**Figure 6:**
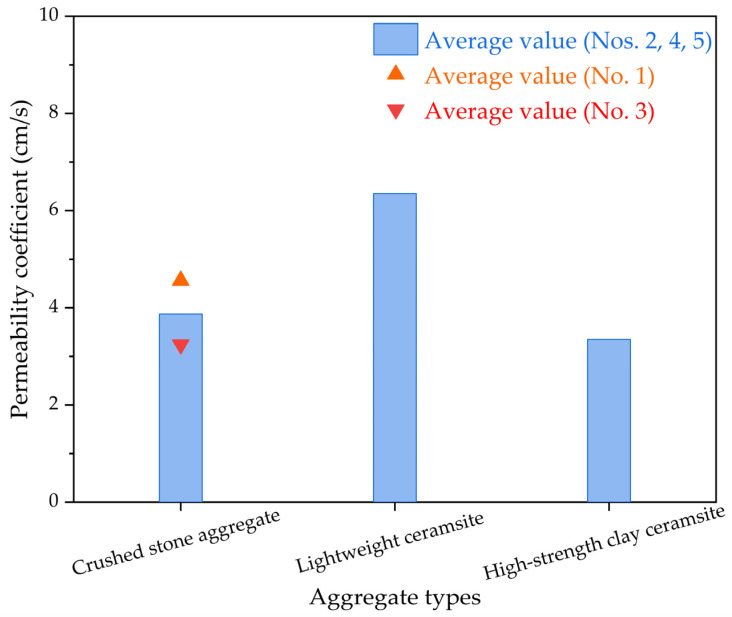
The permeability coefficient results of permeable epoxy resin-based mixtures incorporating various categories of aggregates.

The results of the penetrability characteristics for the permeable epoxy resin-based mixtures incorporating various categories of aggregates are presented in Figure 6. The observed pattern reveals that the permeability coefficient of epoxy resin-based permeable concrete follows the sequence: low-weight ceramsite > ordinary crushed stone aggregate > high-strength clay ceramsite. This phenomenon primarily arises from the influence of pore characteristics, which are dictated through the granule scale as well as the shapeliness of the aggregate itself. As epoxy resin-based permeable concrete solely contains coarse aggregates without any fine aggregates, significant pores exist between the coarse aggregates. The magnitude of porosity primarily depends on the granule scale for coarse aggregates. Notably, the larger the granule scale, the greater the pore characteristics, such as the porosity of permeable epoxy resin-based mixture samples made up of these coarse aggregates, subsequently resulting in a higher permeability coefficient. This view is supported by some strong evidence [41,42,43,44].

### 3.3. Exploration on Compressive Strength Simulation of Epoxy Resin-Based Permeable Concrete with Ceramsite Based on Discrete Element Method

**Figure 7:**
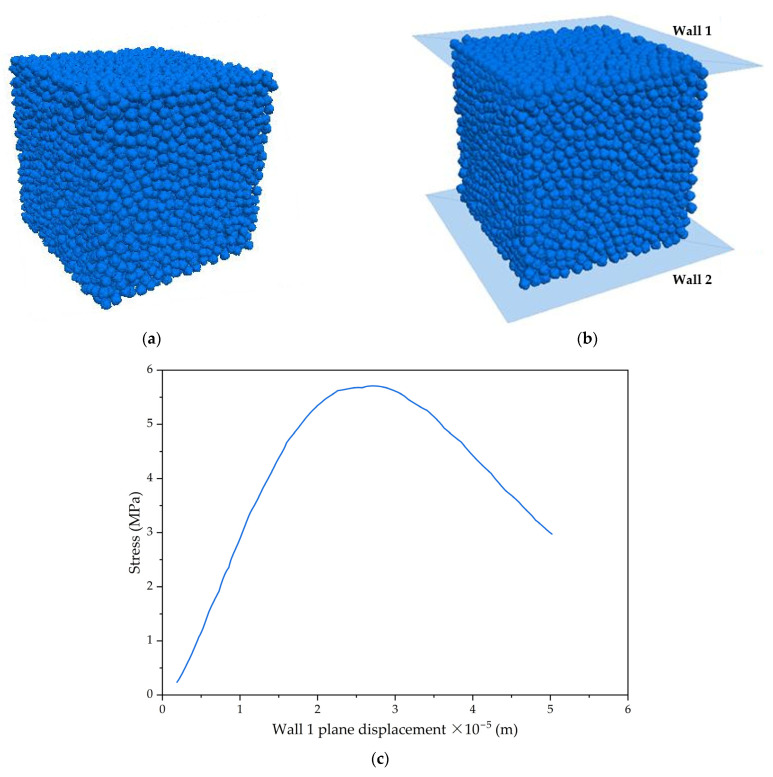
The discrete element analysis of pervious concrete: (**a**) discrete element model; (**b**) compression stress work platform; (**c**) stress-strain displacement curve.

Concrete is a multiphase mixture composed of binder materials, aggregates, sand, and other components mixed together in a certain proportion. Its specific mechanical performance indicators can be determined through processes such as specimen preparation, curing, and testing using testing machines in order to assess its quality. However, this traditional method is characterized by long testing cycles, a high consumption of manpower and resources for specimen preparation, significant dispersion in test results, and a multitude of factors influencing the results. This section focuses on studying the establishment of a discrete element model and conducting a numerical analysis of compression stress for permeable epoxy resin-based mixtures based on a discrete element analysis, aiming to verify the feasibility of using discrete element methods to simulate pervious concrete. Based on the commercial program ABAQUS for the discrete element simulation, the FISH language is employed to write commands to create a model [45]. The model is assigned relevant contact parameters, and the resulting discrete element model of permeable concrete is compared with the laboratory specimens produced in experiments, as shown in Figure 7a. In the permeable concrete specimens with the dimensions 150 mm × 150 mm × 150 mm, the ceramic aggregates were bound together by an epoxy resin. The specific values of the microscale coefficients, such as the bonding strength between ceramic aggregates and the bonding stiffness between them, are listed as follows: elastic modulus = 1.25 × 10^4^ MPa, normal contact modulus = 1.0 × 10^8^ N/m, tangential contact modulus = 2.5 × 10^10^ N/m, normal bond strength = 70 MPa, and tangential bond strength = 40 MPa.

Based on the discrete element model of the permeable epoxy resin-based mixture, the FISH language is used to write commands to remove the contact walls of the permeable concrete model. Working planes wall 1 and wall 2 were generated on the upper and lower exteriors of permeable epoxy resin-based mixtures, respectively. During the course of simulating the compression stress test for the permeable epoxy resin-based mixture, the plane wall 2 remained unchanged, while the descent speed of plane wall 1 was controlled to load the pervious concrete specimen. The compression stress work platform for the permeable epoxy resin-based mixture is displayed in Figure 7b. The loading speed of the upper plane was manipulated to be 2 mm/min. The pressure magnitude on the upper plane and the displacement change of the upper plane were detected to obtain the relationship curve between the stress magnitude on the upper plane and the displacement change of the upper plane, as depicted in Figure 7c. Based on the results from Figure 7, it is obvious that the stress on the working plane wall 1 increases with time until it reaches its peak value, after which it starts to decrease. The compression stress for the permeable epoxy resin-based mixture specimen model was determined to be 5.71 MPa, which is similar to the laboratory-measured compression stress of 5.97 MPa shown in Figure 4. Therefore, the simulated compression stress value is considered to be a true and valid representation. From Figure 7, it can be seen that the pervious concrete presents ductile properties, not brittle ones, which are mainly due to its internal structure. Permeable concrete is a porous and highly permeable material that has a large number of interconnected pores. These pores can effectively absorb and distribute external forces, thereby making the permeable concrete have a certain toughness compared to traditional concrete [46].

### 3.4. Freeze-Thaw Durability Analysis of Epoxy Resin-Based Permeable Concrete with Ceramsite

**Figure 8:**
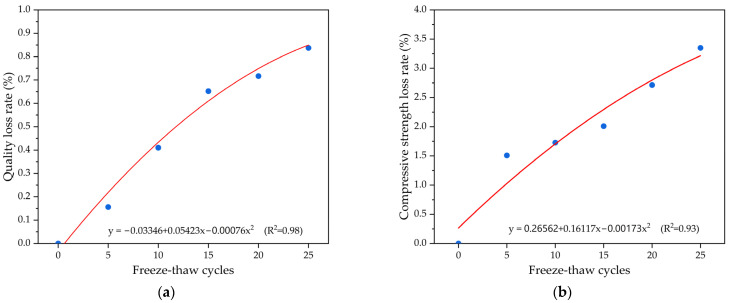
The freeze-thaw durability of a permeable epoxy resin-based mixture incorporating ceramsite: (**a**) weight attrition rate and (**b**) compression stress attrition rate.

Figure 8 depicts the freeze-thaw endurance for permeable epoxy resin-based mixtures incorporating ceramicite by presenting the weight attrition rate as well as the intensity attrition rate separately. As shown in Figure 8a, the weight of the permeable epoxy resin-based mixture samples declines as the number of freeze-thaw loops increases. This deterioration can be attributed to the accumulation of ice formed from the condensation of free water in the voids in the course of the freeze-thaw loops. The increased volume of ice induces damaging stress on the permeable concrete specimens from within. With successive freeze-thaw loops, this disastrous interior traction regularly acts upon the bonding forces between the epoxy resin binder and aggregates for the permeable epoxy resin-based mixture samples, resulting in a decline in the adhesion strength and aggregate detachment. Consequently, the weight of the permeable epoxy resin-based mixture samples exhibited a downward trendline. Upon reaching 25 freeze-thaw cycles, the weight attrition for permeable epoxy resin-based mixture samples amounted to 0.837%. In Figure 8b, it is clear that the compression stress for the permeable epoxy resin-based mixture samples decreases to varying extents as the number of freeze-thaw loop increases. This is attributed to the reduction in adhesion performance between the epoxy resin binder and aggregate, causing certain aggregates to detach from the permeable epoxy resin-based mixture samples. Consequently, all the characteristics of permeable epoxy resin-based mixture samples are significantly affected, giving rise to a diminishment in compression stress. After 25 freezing-thaw loops, the compression stress attrition rate for the permeable epoxy resin-based mixture samples was measured at 3.35%. A quadratic polynomial regression analysis was employed to establish the association between freeze-thaw loops and endurance indices, including the weight attrition rate and compression stress attrition rate for the permeable epoxy resin-based mixture samples. The analysis reveals a strong correlation between the number of freeze-thaw loops and the endurance attrition rate, with an R^2^ value of approximately 0.93.

## 4. Conclusions

In this investigation, the preparation and performance investigation of epoxy resin-based permeable concrete containing ceramsite proves it to be a promising and effective approach in developing sustainable and environmentally friendly construction materials. Through a detailed experimental study and simulation exploration, it has been established that the addition of ceramsite can significantly improve the preparation of permeable concrete, making it suitable for various applications such as pavement and road construction.

(1) The epoxy resin binder–aggregate ratio is used to characterize the self-reinforcement effect of the epoxy resin matrix. As the epoxy resin binder–aggregate proportion grows, the compression stress value for the epoxy resin-based permeable concrete significantly increases. In contrast, the permeability coefficient of epoxy resin-based permeable concrete is inversely proportional to the epoxy resin binder–aggregate ratio; the larger the epoxy resin binder–aggregate ratio, the smaller the permeability coefficient.

(2) The type of aggregate has different effects on the compression stress as well as the permeability coefficient for permeable epoxy resin-based mixtures. Compression stress was primarily supplied through aggregate strength, and pore characteristics was affected by the aggregate granule scale and shapeliness. The high-strength clay ceramsite provided the topmost compression stress, while the low-weight ceramsite had the topmost penetrability characteristics.

(3) Based on the changes in the force on the lower surface, the ultimate load of the discrete element model was determined. The simulated compression stress of the permeable concrete was 5.71 MPa, which is consistent with the measured compression stress of 5.97 MPa, indicating that the discrete element simulation method is effective and feasible for simulating the compression stress of permeable concrete.

(4) A quadratic polynomial regression analysis was utilized to appraise the relationship of endurance and freeze-thaw loops, acquiring an R^2^ value of near 0.93. After 25 freeze-thaw loops, the permeable epoxy resin-based mixture samples displayed only a miniature zone of detachment of ceramic granules on the outside.

In conclusion, the investigation on epoxy resin-based permeable concrete containing ceramsite demonstrates its potential as a viable alternative to traditional concrete materials. Its sustainability and environmental benefits make it an attractive option for future construction projects. Further research and development in this field are recommended to explore its full potential and fine-tune its performance for specific applications. If the application of epoxy resin-based permeable concrete is intended for water collection, the further study should include measuring the thermal properties with the focus on frost or daily temperature change.

## Figures and Tables

**Table 1:** The properties of the used modified epoxide resin binder.

Properties	Density (g/cm^3^)	Setting Time (h)		Tensile Strength (MPa)	Shear Strength (MPa)
		Initial	Final		
Test	1.5	0.5	1.5	70.5	41.3

**Table 2:** The physical characteristics for three categories of aggregates in this investigation.

Type	Density (g/cm^3^)	Bulk Density (g/cm^3^)	Crushing Value (%)	Strength (MPa)
Normal	2.32	1.44	11.3	4.8
Low-weight ceramsite	0.37	0.22	86.4	0.12
High-strength clay ceramsite	1.80	1.10	10.8	5.2

**Table 3:** The pervious concrete mix proportioning data in this study.

Aggregate Type		Epoxy Resin Binder/ Aggregate Ratio	Epoxy Resin Binder (kg)	Aggregate (kg)	Porosity (%)
Ordinary crushed stone	No. 1	3%	43.2	1440	32.6
	No. 2	4%	57.6	1440	31.9
	No. 3	5%	72	1440	30.7
Lightweight ceramsite	No. 4	4%	57.6	220	33.7
High-strength clay ceramsite	No. 5	4%	57.6	1100	31.6

## Data Availability

Data are contained within the article.
